# Simplified aluminum nitride processing for low-loss integrated photonics and nonlinear optics

**DOI:** 10.1038/s44310-026-00107-7

**Published:** 2026-03-04

**Authors:** Haochen Yan, Shuangyou Zhang, Arghadeep Pal, Alekhya Ghosh, Abdullah Alabbadi, Masoud Kheyri, Toby Bi, Yaojing Zhang, Irina Harder, Olga Ohletz, Florentina Gannott, Alexander Gumann, Eduard Butzen, Katrin Ludwig, Pascal Del’Haye

**Affiliations:** 1 Max Planck Institute for the Science of Light, Erlangen, Germany; 2 Department of Physics, Friedrich-Alexander-Universität Erlangen-Nürnberg, Erlangen, Germany; 3 Department of Electrical and Photonics Engineering, Technical University of Denmark, Kongens Lyngby, Denmark; 4 School of Science and Engineering, The Chinese University of Hong Kong (Shenzhen), Shenzhen, Guangdong China

**Keywords:** Microresonators, Optics and photonics, Nonlinear optics

## Abstract

Aluminum nitride (AlN) is an extremely promising material for integrated photonics because of the combination of strong χ^(2)^ and χ^(3)^ nonlinearities. However, the intrinsic hardness of the material and charging effects during electron beam lithography make AlN nanofabrication a challenging process. Conventional approaches often require multiple hard masks and a metal mask to fabricate nanostructures. In this letter, we report a novel, simple method to fabricate AlN microresonators by using a single layer of silicon nitride mask combined with a thin conductive polymer layer. The conductive layer can be conveniently removed during developing without requiring an additional etching step. We achieve a high etching selectivity of 4:1 between AlN and the mask, enabling mid-infrared photonic device fabrication, as well as high intrinsic quality (*Q*) factors of up to 1.0 × 10⁶ in AlN microresonators. Furthermore, we demonstrate several nonlinear phenomena within these devices, including frequency comb generation, Raman lasing, third-harmonic generation, and supercontinuum generation.

## Introduction

Aluminum nitride (AlN) has gained significant attention in the field of integrated photonics for its unique properties including a large bandgap (up to 6.2 eV)^1–3^, strong χ^(2)^ nonlinearity^4–6^, high thermal conductivity and significant piezoelectric effect^7–9^, which are typically not accessible in conventional silicon-based materials such as silica and silicon nitride. Leveraging these properties, AlN gained lots of interest for applications like second harmonic generation^10–13^, Pockels-effect-based modulators^14^ and photodetectors in the ultraviolet and near-infrared regimes^15–17^. In addition, as a member of the III-nitride-family materials, AlN shares a similar refractive index, nonlinear index, and strong χ^(3)^ nonlinearity with Si_3_N_4_^3,18^. As a result, integrated photonics devices based on AlN can be extremely versatile and robust for many integrated photonic applications^19–31^.

In the past decades, different fabricating methods for high-quality (*Q*) factor AlN microresonators have been reported. However, all these methods require multiple layers of hard masks combined with a metal mask^32–36^. This complexity arises from the extremely high hardness of the material and thus low etching selectivity between AlN and photoresist, which requires multiple layers of hard masks to attain the desired etching depth. Additionally, strong charging of the material from electron beam (e-beam) lithography requires a conductive material (metal mask, typically Ti/Cr/Au) to avoid stitching errors and misalignment issues.

In this work, we report a novel fabrication process that utilizes a single hard mask layer without requiring any metal mask on top. To address the charging problem associated with e-beam lithography, we use a water-solvable conductive layer, which can be easily removed during the development step, simplifying the overall fabrication process. The etching selectivity between AlN and the mask is as high as 4:1, which enables very deep AlN etching that can be used for mid-infrared devices based on thick AlN layers (>2 µm) without modifying the recipe. Our devices are fabricated using single-crystalline AlN on sapphire^36^. The fabricated AlN microresonators have a high intrinsic *Q* factor of around 1.0 × 10^6^ in the telecom band, enabling the successful generation of Kerr frequency combs around 1550 nm. Additionally, other nonlinear phenomena, such as Raman lasing, third harmonic generation (THG), and supercontinuum generation are also observed, which further verifies the suitability of the fabricated devices for nonlinear optics applications.

## Results

### Device fabrication and characterization

**Fig. 1:**
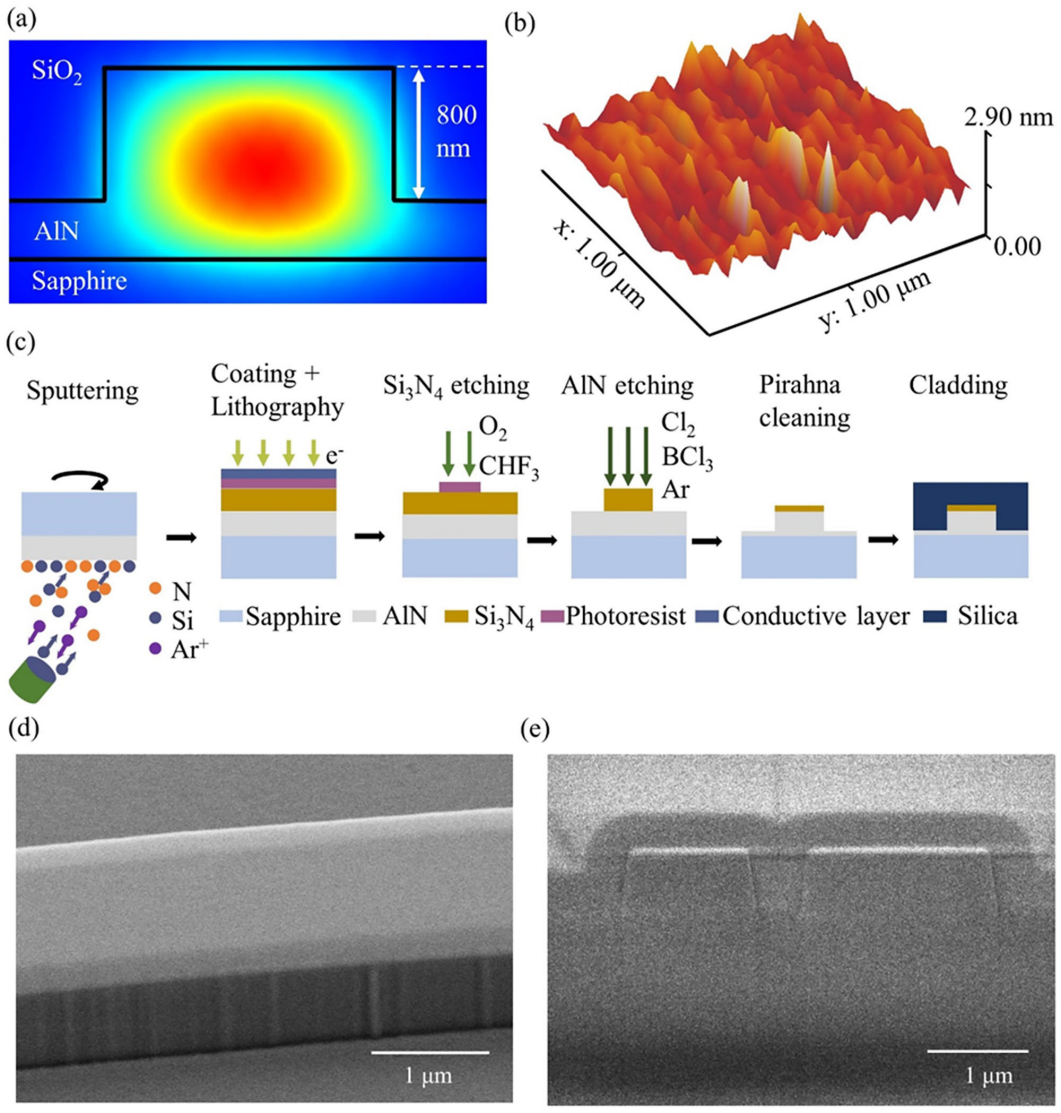
Metal-mask free AlN microresonator fabrication. **a** Simulation result of the mode profile for a partially-etched ring microresonator (~800 nm etching depth). **b** AFM image of a single crystalline AlN-on-sapphire sample with a 250-nm-thick Si_3_N_4_ mask on top. **c** Fabrication flow for the AlN process. **d** SEM image of an AlN waveguide. **e** FIB-SEM cross section of the coupling region between ring and waveguide.

To fabricate the low-loss AlN nanophotonic devices, we start with a commercial AlN-on-sapphire wafer (DOWA) with a single crystalline 1-µm-thick layer of AlN, where the substrate is c-plane sapphire. This thickness ensures high crystalline quality^37^. To achieve good mode confinement and anomalous dispersion in the telecom band, we first determine the desired etching depth by simulating the mode profile, as shown in Fig. 1a. We find that around 800 nm etching depth is enough for light confinement and achieving anomalous dispersion. Next, we evaluate the wafer surface roughness with an atomic force microscope (AFM), yielding a root mean square roughness of ~0.34 nm, as depicted in Fig. 1b. The fabrication flow for realizing an AlN microresonator is illustrated in Fig. 1c. We begin with reactive magnetron sputtering of a 250-nm-thick Si_3_N_4_ hard mask onto the AlN layer at room temperature^38^. This thickness of the hard mask is sufficient to survive ~800 nm AlN etching. To pattern the photonic structures, a layer of negative photoresist (ma-N 2405) is spin coated onto the Si_3_N_4_. In order to avoid charging effects, we spin-coat a conductive layer (mr-Conductive) on top of the photoresist. This water-solvable layer is later removed together with the photoresist, during the development step using developer ma-D 532, without requiring additional treatment. The pattern is then transferred to the Si_3_N_4_ mask by inductively coupled plasma reactive ion etching, using a gas mixture of CHF_3_ and O_2_. The AlN layer is then etched using a Cl_2_/BCl_3_/Ar gas mixture at a ratio of 25:6:9, achieving an etching depth of around 800 nm. The etching selectivity between AlN and Si_3_N_4_ is ~4:1, which is sufficient for our process. In comparison, we measured an etching selectivity of 1:3.2 for AlN with respect to a photoresist (ma-N 2405) mask. The Si_3_N_4_ mask is almost completely removed during the AlN etching process, leaving only a very thin layer (tens of nm) of Si_3_N_4_ on top of the AlN. Given the similar refractive indices of Si_3_N_4_ and AlN, this thin Si_3_N_4_ layer does not influence the mode profile as well as the dispersion significantly and can be kept on the photonic structures (see “Methods”). For precise dispersion engineering applications, the sputtered Si_3_N_4_ layer thickness needs to be controlled so that the mask is completely removed during the AlN etching. In this case, the dispersion will not be influenced by the remaining Si_3_N_4_ layer. Finally, the sample undergoes cleaning with Piranha solution to remove any residual photoresist. A scanning electron microscope (SEM) image of the fabricated structure after AlN etching, shown in Fig. 1d, reveals very small sidewall roughness.

To protect the photonic structures, a ~3-µm-thick SiO_2_ cladding layer is deposited onto the chip. We perform a two-step silica deposition to avoid air voids between bus waveguide and resonator waveguide. The first 400 nm oxide is deposited using atomic layer deposition (ALD) and then another layer of 2.5 μm silica is deposited by plasma enhanced chemical vapor deposition. A Focused ion beam (FIB) SEM image of the coupling region between waveguide and ring resonator is shown in Fig. 1e, where we can confirm that the gap between the ring and waveguide is filled properly by the ALD silica. Additionally, we can identify the AlN etching angle of ~81°. No annealing is performed after encapsulation. Finally, the sample is diced for edge coupling from a fiber to the bus waveguide. It should be noted that the sapphire substrate is extremely hard, making cutting the whole chip challenging and resulting in chipped edges. Instead, we partially saw cut into the sapphire side, followed by manually breaking the chip.

**Fig. 2:**
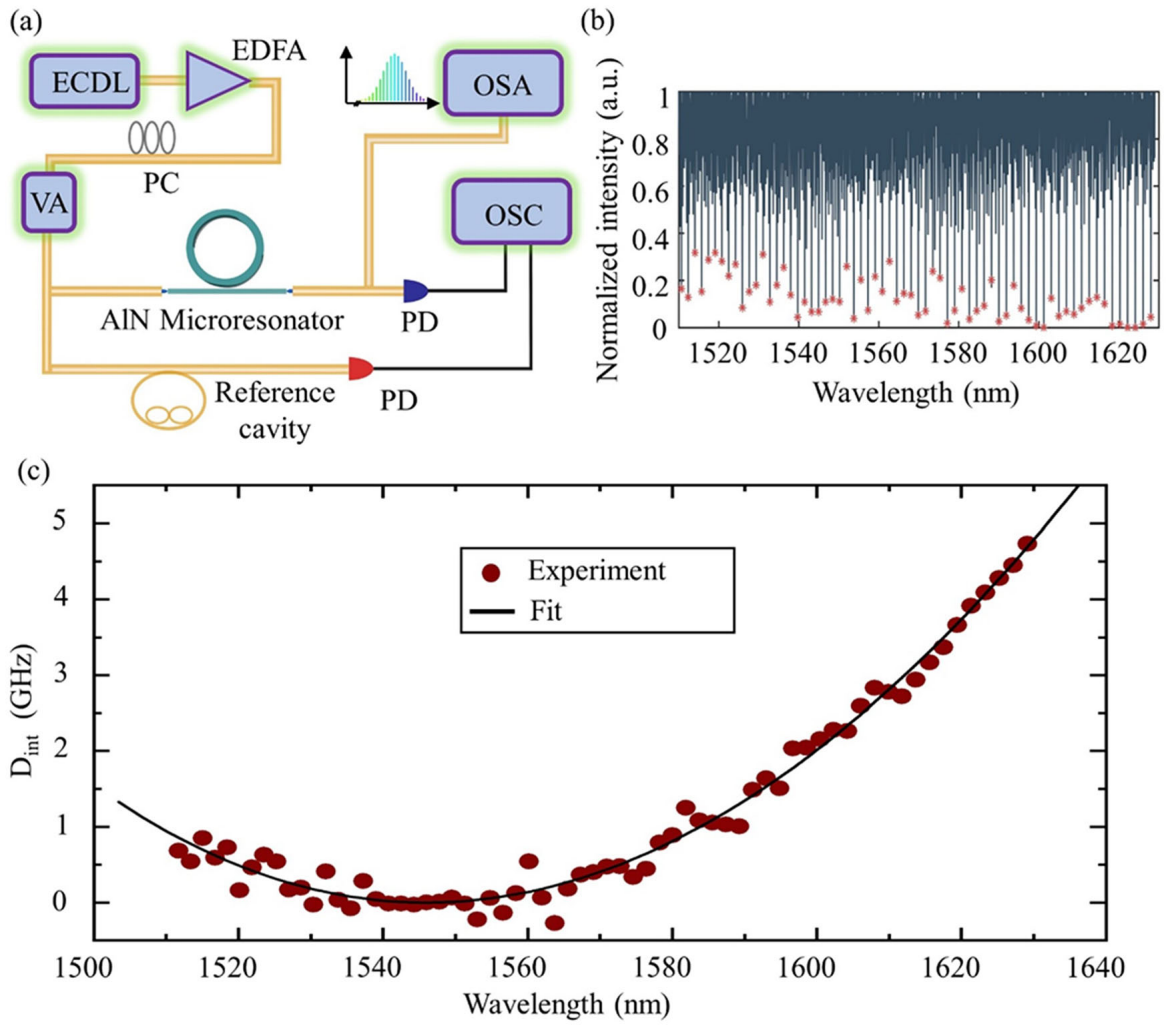
Experimental setup and device characterization. **a** Setup for characterization of the photonic chip and generating different types of nonlinear processes in AlN devices. ECDL external cavity diode laser, EDFA Erbium-doped fiber amplifier, PC polarization controller, VA Variable attenuator, PD photodiode, OSC oscilloscope, OSA optical spectrum analyzer. **b** Resonance spectrum across the C-band and L-band. **c** Measured dispersion profile at the pump wavelength of 1546 nm and a corresponding second-order polynomial fit.

The fabricated devices are characterized with high-precision tunable diode laser spectroscopy^39^ using the setup shown in Fig. 2a. We use an external cavity diode laser in the C- and L-band to probe the AlN devices. Light is coupled in and out of the chip via edge coupling with two lensed fibers. The light polarization is controlled by a polarization controller to match either the quasi-transverse magnetic (TM) or the quasi-transverse electric (TE) modes of the waveguides. The measured total insertion loss from fiber to fiber is around 8 dB. The laser power in these measurements is sufficiently low to avoid Kerr and thermal effects. During frequency scanning, the laser frequency can be precisely determined by a fiber reference cavity calibrated by dual radio frequency modulation^39^. The transmission spectra of the AlN resonators and the reference cavity are recorded simultaneously. Figure 2b shows the transmission spectrum of the AlN ring resonator with 100-micron radius and 1.8-micron waveguide width. Using the measured resonance frequencies, we can calculate the dispersion profile of the device. The resonance frequencies are described as a function of the integrated dispersion^40^ from the Taylor expansion:

$${\omega }_{k}={\omega }_{0}+{D}_{1}k+{D}_{\mathrm{int}}(k).$$

In the above formula, *ω*_*k*_ are the resonance frequencies with mode number *k* (with *k* = 0 being an arbitrarily chosen center mode). *D*_1_ indicates the free spectral range (FSR) at the center mode such that *D*_1_ = 2π × FSR, and *D*_int_ represents the integrated dispersion. The measured dispersion and the polynomial fits are shown as dark-red circles and black lines respectively in Fig. 2c. Our sample exhibits anomalous dispersion, which agrees with our simulation results (see “Methods”). This dispersion regime is important for the generation of bright solitons^41,42^.

**Fig. 3:**
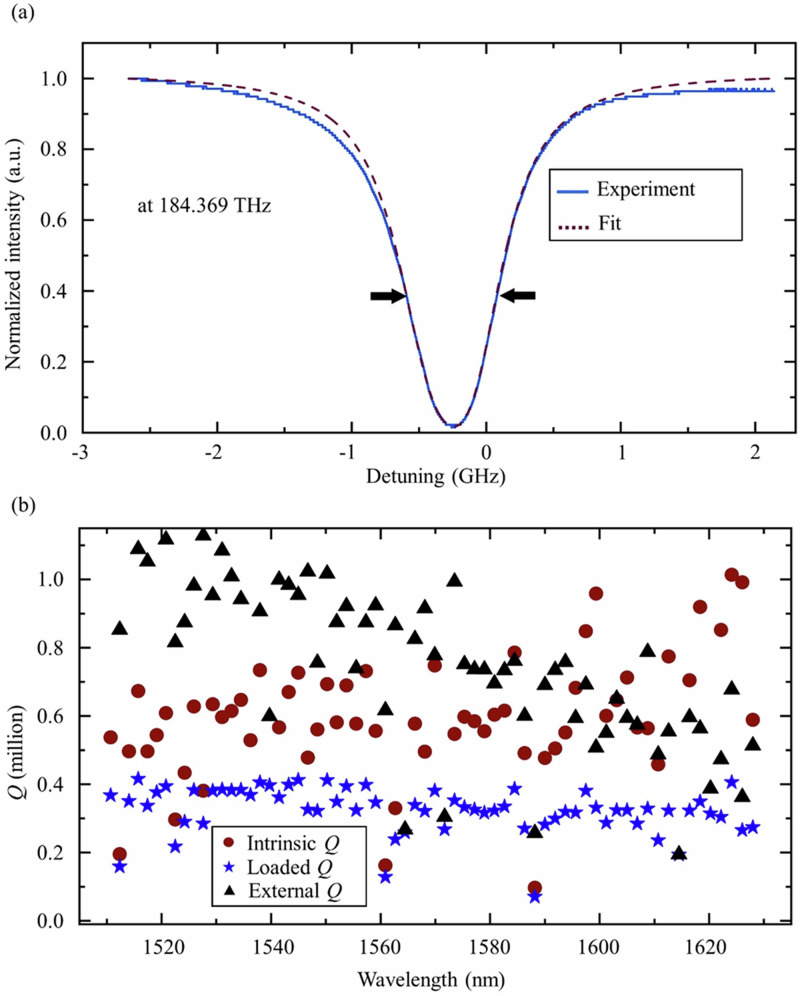
*Q* factor measurements of the fabricated microresonators. **a** Normalized transmission spectrum for a high *Q* mode. **b** Summarized loaded, intrinsic, and external *Q* factors across the C-band and L-band.

The *Q* factors of the resonators are shown in Fig. 3. Figure 3a shows a selected high *Q* resonance around 1626 nm. The fitted linewidth of the resonance is around 694 MHz, with a loaded *Q* of 0.27 million. The intrinsic *Q* is calculated to be ~1.0 million, considering slight over-coupling of the bus waveguide to the resonator. Using the intrinsic *Q* value and a FSR of around 221 GHz, the propagation loss can be determined using the formula: *l* = *f*_r_/(*R* × FSR × *Q*_i_), where *f*_r_ indicates the resonance frequency and *R* is the ring resonator radius. Based on these parameters, the propagation loss is calculated to be 0.36 dB/cm. Figure 3b shows a measurement of all the *Q* factors of the resonances in the range between 1510 nm and 1630 nm. The average intrinsic *Q* is 0.65 million.

In the next sections, we investigate the low loss AlN platform for nonlinear optical effects, including Kerr frequency comb generation, Raman lasing, THG and supercontinuum generation. These phenomena have significant potential for various applications with integrated photonic circuits based on AlN.

### Kerr frequency comb generation

**Fig. 4:**
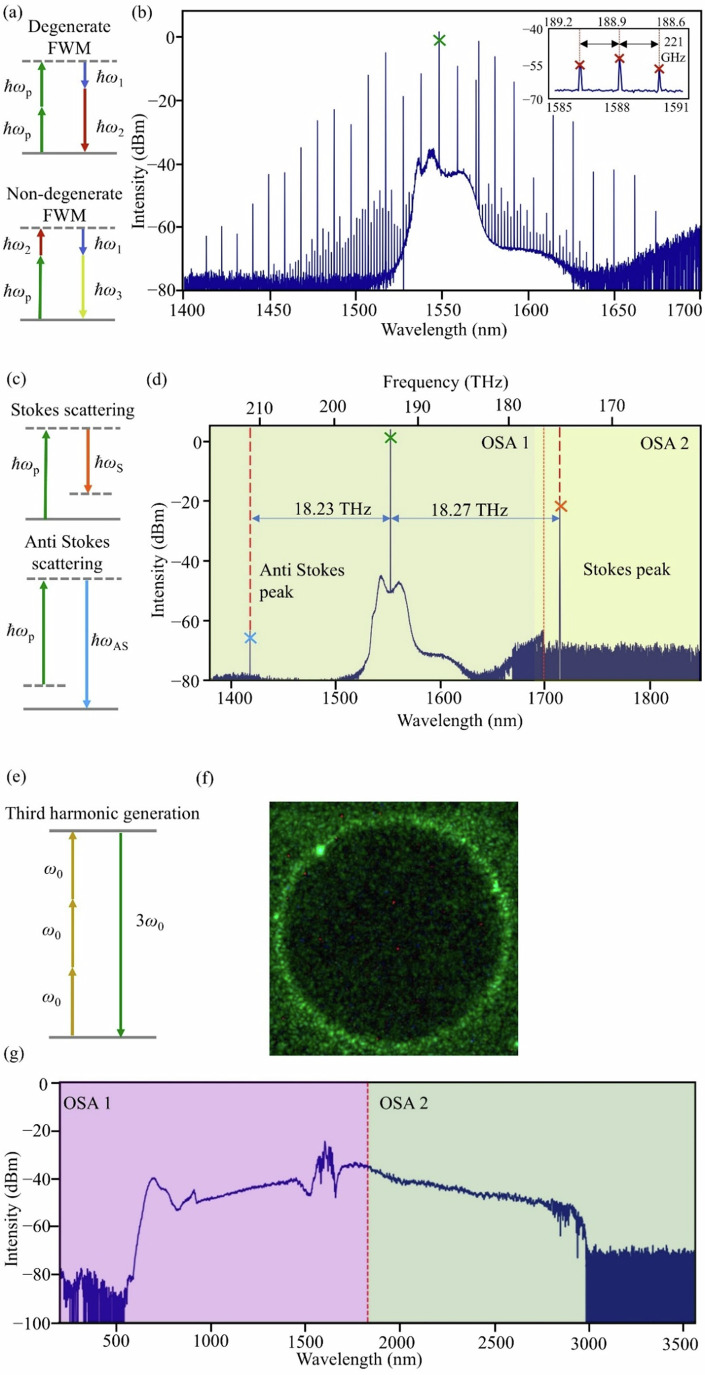
Various nonlinear effects in AlN. **a** Energy diagram for frequency comb generation through four-wave mixing. **b** Measured comb spectrum. **c** Energy diagram for Raman scattering. **d** Measured Stokes and anti-Stokes sidebands. **e** Energy diagram for third-harmonic generation. **f** Third harmonic light emission from ring resonator. **g** Supercontinuum generation covering ~2.5 octaves from 600 nm to 2.9 µm.

Figure 4a, shows degenerate and non-degenerate four-wave mixing (FWM) processes resulting in the generation of a frequency comb spectrum. In the experiment, we pump the resonator with 800 mW on-chip power and tune the pump laser frequency into a resonance from the blue detuned side. The spectrum of the Kerr frequency comb is recorded by an optical spectrum analyzed (OSA) and shown in Fig. 4b, where the pump laser is marked with a green cross. The comb lines span around 250 nm at an FSR of 221 GHz.

### Raman lasing

A simplified Raman lasing scheme is illustrated in Fig. 4c, where the incident pump photon interacts with a phonon through inelastic scattering. More specifically, the incident photon can transfer its energy to the vibrational mode, and thus the emitted photon has lower energy and longer wavelength (Stokes shift). Alternatively, the incident photon can also gain energy from the excited vibrational mode, and thus the resulting emission has higher energy and shorter wavelength (anti-Stokes shift). Since the number of molecules in the vibrational ground state have a much bigger population than the excited ones, the power of the Stokes sideband is much stronger than the power in the anti-Stokes sideband. The wurtzite structure of AlN supports six different types of Raman phonons. In the experiment, we first align the polarization of the pump laser to the TM mode that is aligned with the AlN c-axis, then tune the laser frequency from the blue-detuned side into the resonance. When the intra-cavity power gradually increases, we observe a Raman Stokes signal at 1715.3 nm and an anti-Stokes signal at 1419.2 nm as shown in Fig. 4d. The Raman shift with respect to the pump laser is ~18.2 THz. This shift corresponds to an A_1_^TO^ phonon^31^.

### Third harmonic generation

The strong χ^3^ nonlinearity of AlN supports THG, as depicted in Fig. 4e. By pumping a mode at 1538.2 nm, we observe the emission of strong green light. The conversion of light from telecom regime to the infrared regime is captured by a visible camera as shown in Fig. 4f. The phase matching of the THG process happens at fixed detuning values of the pump laser with respect to the resonator mode and is determined by the resonator dispersion.

### Supercontinuum generation

Finally, we demonstrate supercontinuum generation in an AlN waveguide, which is a mediated by a combination of nonlinear phenomena, including FWM, self-phase modulation, cross-phase modulation and harmonic generation. A 50-femtosecond pulse train from an Er-fiber frequency comb with 100 MHz repetition rate and 1560 nm central wavelength is used to pump the nonlinear waveguide. The pulses are coupled into the bus waveguide using an aspheric lens (0.6 numerical aperture) with an estimated insertion loss of −5 dB/facet. Driven by the femtosecond pulse excitation, the supercontinuum emerges as a broadband spectrum resulting from the interplay of nonlinear processes as well as optimized dispersion. We observe a two-octave spectrum from VIS to Mid-IR, which is collected by an InF_3_ fiber, shown in Fig. 4g. The average on-chip power is around 10 mW. The large spectral broadening takes advantages of both the large Kerr nonlinearity and high confinement of the AlN waveguide and has significant potential for applications in optical frequency metrology, high-resolution spectroscopy and optical telecommunications.

## Discussion

In conclusion, we demonstrate a simple fabrication process for low-loss AlN-based nanophotonics without the requirement for metal masks and thermal annealing. The etching selectivity between AlN and the Si_3_N_4_ mask has an unprecedentedly high ratio of 4:1. With this process we achieve *Q-*factors of 1.0 million in AlN microresonators with a corresponding propagation loss of 0.36 dB/cm. Utilizing our fabricated AlN devices, we demonstrate Kerr frequency combs, Raman lasing, THG and supercontinuum generation. AlN as an integrated photonics platform has a lot of potential for a large variety of applications, including coupled microresonator systems for optical data processing and frequency combs with tailored dispersion. Given the presence of both second and third order nonlinearities, this platform can be used for cascaded nonlinear phenomena with hybrid nonlinear interactions^43^. Additional applications are Brillouin frequency combs^44^, spontaneous symmetry-breaking^45^ and visible photonics^46^. In addition to these applications, the high etching selectivity of our recipe can realize deep etching without adding any extra steps except for increasing the sputtered Si_3_N_4_ layer thickness. 3 microns of AlN etching can be achieved by using less than 800 nm of Si_3_N_4_ mask. Thus, our recipe can extend the wavelength range for AlN devices to the mid-infrared, where thicker waveguides are required^47^. These research directions can further unlock the potential of the AlN platform for emerging applications in integrated nonlinear optics and quantum photonics.

## Methods

**Fig. 5:**
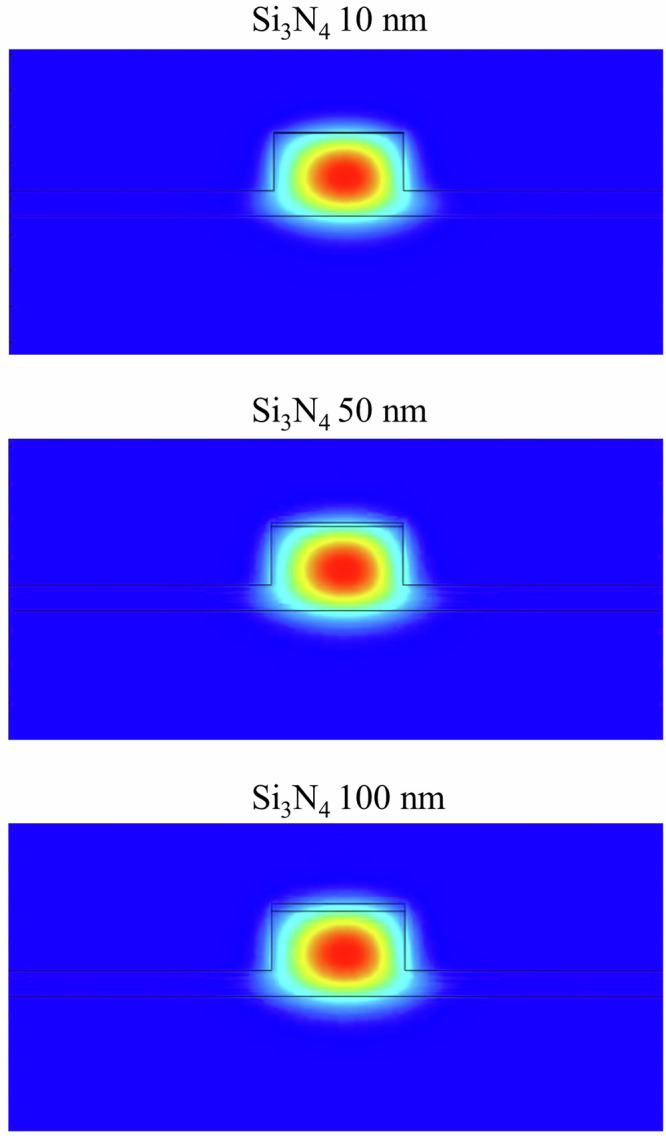
Simulation results for the mode profile of an AlN microresonator with a thin Si_3_N_4_ layer on top. The mode profile is plotted for a wavelength of 1546 nm. The Si_3_N_4_ layer thickness is changed between 10 nm to 100 nm (top to bottom). The change in Si_3_N_4_ layer thickness has no significant impact on the mode profile.

**Fig. 6:**
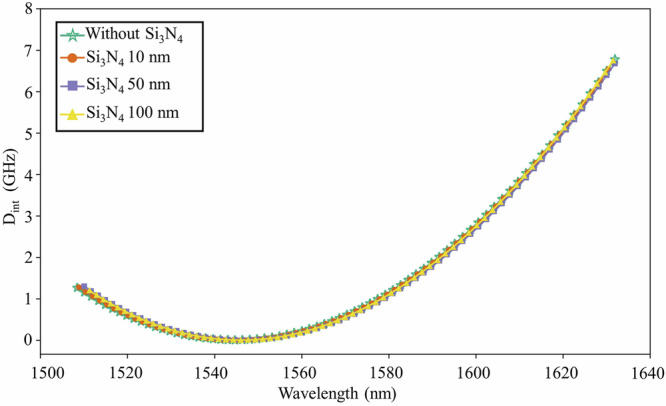
Simulation results of the integrated dispersion Dint of the AlN microresonator. The pump wavelength is 1546 nm. The Si_3_N_4_ layer thickness is changed between 0 nm and 100 nm. No significant change of the dispersion profile is observed as a result of the thin Si_3_N_4_ layer.

During the fabrication process a 250-nm-thick Si_3_N_4_ hard mask was used for pattern transfer. Instead of removing this layer in an additional post-etch cleaning step, we verified its influence on the optical properties of the device through finite element simulations. Based on the structure shown in Fig. 1a, we add another Si_3_N_4_ layer in between the AlN layer and the cladding layer. The Si_3_N_4_ layer thickness is systematically changed from 0 nm to 100 nm (a very conservative upper bound). We first investigate the mode confinement with the results shown in Fig. 5. We identify that the mode shape remains unchanged when we increase the Si_3_N_4_ layer thickness. Thus, the results here confirm that the optical mode remains tightly confined within the AlN core with negligible distortion even at the maximum simulated thickness. We then investigate how the dispersion profile is influenced by the presence of the residual Si_3_N_4_ layer. We first simulate the dispersion without including the Si_3_N_4_ layer and then add a mask layer and change its thickness between 10 nm and 100 nm, as shown in Fig. 6. We observe anomalous dispersion, which agrees with the experimental results. The dispersion profile does not show a significant change in the presence of the Si_3_N_4_ layer across the simulated wavelength range. These results show that the residual Si_3_N_4_ layer, if left in place, does not degrade the mode confinement or the dispersion characteristics of the device. This finding supports our decision to omit the mask removal step, which simplifies the overall fabrication process and minimizes the risk of introducing surface roughness or etching-induced damage.

## Data Availability

The data supporting this study are available upon reasonable request.
